# Skeletal Muscle Expression of the Adhesion-GPCR CD97: CD97 Deletion Induces an Abnormal Structure of the Sarcoplasmatic Reticulum but Does Not Impair Skeletal Muscle Function

**DOI:** 10.1371/journal.pone.0100513

**Published:** 2014-06-20

**Authors:** Tatiana Zyryanova, Rick Schneider, Volker Adams, Doreen Sittig, Christiane Kerner, Claudia Gebhardt, Henrik Ruffert, Stefan Glasmacher, Pierre Hepp, Karla Punkt, Jochen Neuhaus, Jörg Hamann, Gabriela Aust

**Affiliations:** 1 Department of Surgery, Research Laboratories, University of Leipzig, Leipzig, Germany; 2 Heart Center Leipzig, University of Leipzig, Leipzig, Germany; 3 Department of Anaesthesiology and Intensive Care Medicine, University of Leipzig, Leipzig, Germany; 4 Clinic for Trauma and Reconstructive Surgery, University of Leipzig, Leipzig, Germany; 5 Institute of Anatomy, University of Leipzig, Leipzig, Germany; 6 Clinic of Urology, Research Laboratories, University of Leipzig, Leipzig, Germany; 7 Department of Experimental Immunology, Academic Medical Center, University of Amsterdam, Amsterdam, The Netherlands; University of Debrecen, Hungary

## Abstract

CD97 is a widely expressed adhesion class G-protein-coupled receptor (aGPCR). Here, we investigated the presence of CD97 in normal and malignant human skeletal muscle as well as the ultrastructural and functional consequences of CD97 deficiency in mice. In normal human skeletal muscle, CD97 was expressed at the peripheral sarcolemma of all myofibers, as revealed by immunostaining of tissue sections and surface labeling of single myocytes using flow cytometry. In muscle cross-sections, an intracellular polygonal, honeycomb-like CD97-staining pattern, typical for molecules located in the T-tubule or sarcoplasmatic reticulum (SR), was additionally found. CD97 co-localized with SR Ca^2+^-ATPase (SERCA), a constituent of the longitudinal SR, but not with the receptors for dihydropyridine (DHPR) or ryanodine (RYR), located in the T-tubule and terminal SR, respectively. Intracellular expression of CD97 was higher in slow-twitch compared to most fast-twitch myofibers. In rhabdomyosarcomas, CD97 was strongly upregulated and in part more N-glycosylated compared to normal skeletal muscle. All tumors were strongly CD97-positive, independent of the underlying histological subtype, suggesting high sensitivity of CD97 for this tumor. Ultrastructural analysis of murine skeletal myofibers confirmed the location of CD97 in the SR. CD97 knock-out mice had a dilated SR, resulting in a partial increase in triad diameter yet not affecting the T-tubule, sarcomeric, and mitochondrial structure. Despite these obvious ultrastructural changes, intracellular Ca^2+^ release from single myofibers, force generation and fatigability of isolated soleus muscles, and wheel-running capacity of mice were not affected by the lack of CD97. We conclude that CD97 is located in the SR and at the peripheral sarcolemma of human and murine skeletal muscle, where its absence affects the structure of the SR without impairing skeletal muscle function.

## Introduction

CD97 is a member of the adhesion-class of G-protein coupled receptor (aGPCR), which are non-canonical seven-transmembrane (7TM) molecules with essential roles in planar cell and tissue polarity, development, tumorigenesis, and immunity [1], [2]. aGPCRs possess a unique structure with an unusual, large extracellular part containing various protein domains and a juxtamembranous GPCR autoproteolysis-inducing (GAIN) domain [2]. The GAIN domain facilitates cleavage into an extracellular N-terminal fragment (NTF) and a C-terminal fragment (CTF) containing the 7TM and the intracellular domain (ICD). Both fragments, i.e. the NTF and CTF, remain non-covalently associated during expression at the cell surface [3], [4].

CD97 is one of the most widely distributed aGPCRs, expressed by all leukocytes and various normal and malignant epithelial cells [5]–[9]. Alternative splicing results in human CD97 isoforms with three to five EGF-like domains in its NTF [10]. On immune cells, CD97 facilitates cell and extracellular matrix interactions [11]–[15]. In normal epithelial cells, CD97 is located in lateral cells contacts and involved in their formation or stabilization [16]. CD97 is upregulated in several carcinomas, where it probably increases tumor cell migration [6], [8], [17].

Initially, expression of CD97 on smooth muscle and, as shown here, skeletal muscle, was overlooked due to the absence of an N-glycosylation-dependent epitope within the EGF-like domains of the NTF that is recognized by several original CD97 antibodies (Abs) [5], [18], [19]. Missing N-glycosylation has functional consequences: “naked” CD97 cannot bind CD55, a cellular ligand essential for leukocyte adhesion and the regulation of CD97 expression in circulation [11], [19], [20]. Interestingly, in most leiomyosarcomas, CD97 becomes partially N-glycosylated and thus stains positive in immunohistology with the original CD97 Abs [21].

Expression of CD97 in normal and malignant smooth muscle raised questions regarding the presence of CD97 in skeletal muscle and its functional consequences. Skeletal muscle fibers are highly organized multicellular structures. Sarcomeres, the contractile units of striated muscles, undergo contractions triggered by calcium release from the sarcoplasmatic reticulum (SR) in response to plasma membrane depolarization [22]. This process is referred to as excitation–contraction coupling and takes place at specialized junctions, the calcium-release units. In skeletal muscle, such units consist of the junctional SR and the transverse (T)-tubules, deep invaginations from the cell surface, forming triads. For calcium release, dihydropyridine receptors (DHPRs) at the T-tubule serve as voltage sensors, while ryanodine receptors (RyRs) and inositol trisphosphate receptor (IP_3_) receptors, located in the membrane of the SR terminal cisternae and in the longitudinal SR, respectively, act as calcium release channels [22]. Uptake of calcium into the SR following release during excitation–contraction coupling is mediated mostly by sarco(endo)plasmatic reticulum Ca^2+^-ATPases (SERCAs), located in the membrane of both terminal cisternae and longitudinal SR. SERCA2a is typical for slow-twitch skeletal and heart muscles, positive for human myosin heavy chain (MHC) type I (MHC^slow^) [23]. SERCA1 is present in fast-twitch fibers positive for MHC IIa or MHC IIX (MHC^fast^). Beside calcium release and reuptake, the SR provides feedback control to balance calcium storage through calcium-binding proteins [22].

Here, we demonstrate that human skeletal muscle and their malignant entities, rhabdomyosarcomas, are CD97-positive, and report the subcellular localization of CD97 within the SR and at the peripheral sarcolemma. CD97-deficient murine myofibers show remarkable ultrastructural changes in the SR that, however, do not result in obvious adverse effects in intracellular calcium release, muscle force generation and fatigability of soleus muscles, as well as mice wheel-running capacity.

## Materials and Methods

### Ethics statement

This research complies with the ethics guidelines of the University of Leipzig. We obtained approval from the Ethics Committee of the Medical Faculty of the University of Leipzig (numbers 028-2000, 042-2001, 111-2004) to analyze normal and malignant human muscle samples and written informed consent from all patients involved in this study. For mouse wheel running experiments, we obtained ethics approval from the Landesdirektion Leipzig (TVV16/11).

### Human tissues

Biopsies of vastus medialis muscles were obtained from healthy donors after a negative *in vitro* contracture test to diagnose susceptibility to malignant hyperthermia. Small samples of human spinalis muscle were taken during dorsal stabilization after lumbar spine fractures. Samples of healthy semitendinosus muscles were taken during reconstruction operation of anterior cruciate ligament rupture by semitendinosus tendon autograft. Rhabdomyosarcomas were obtained after tumor resection.

### Mice

*CD97*-deficient (CD97Ko) mice have been generated recently [9]. CD97^tm1Dgen^ (CD97LacZ knock-in) mice, in which the endogenous CD97 promoter drives nuclear expression of β-galactosidase, were obtained from The Jackson Laboratory (Bar Harbor, ME, USA). Histochemical detection of β-galactosidase activity was performed as described [9]. All mouse strains and the corresponding C57BL/6J wild-type (WT) littermates were housed at the Medical Experimental Center of the University of Leipzig.

### Cells and cell lines

Normal human skeletal muscle cells were obtained from PromoCell GmbH (Heidelberg, Germany). The human rhabdomyosarcoma cell line Hs729.T and the murine cell lines RAW 264.7 (monocyte/macrophage), C2C12 (myoblastic), 3H3/10T1/2 and 3T3 (fibroblastic), and CMT-93 (rectal carcinoma) were purchased from the American Type Culture Collection (ATCC, Rockville, MD, USA). C2C12 myoblasts were differentiated into myocytes by culture with 2% horse serum over 96 h. Murine CT-26 (colorectal carcinoma) cells were kindly provided by Dr. V. Schirrmacher (German Cancer Research Center, Heidelberg, Germany). Murine Hep 53.4 (hepatocyte) cells were obtained from The Deutsche Tumorbank (German Cancer Research Center, Heidelberg, Germany). Human peripheral blood lymphocytes were obtained by density gradient centrifugation [5].

### Antibodies

**Figure 1:**
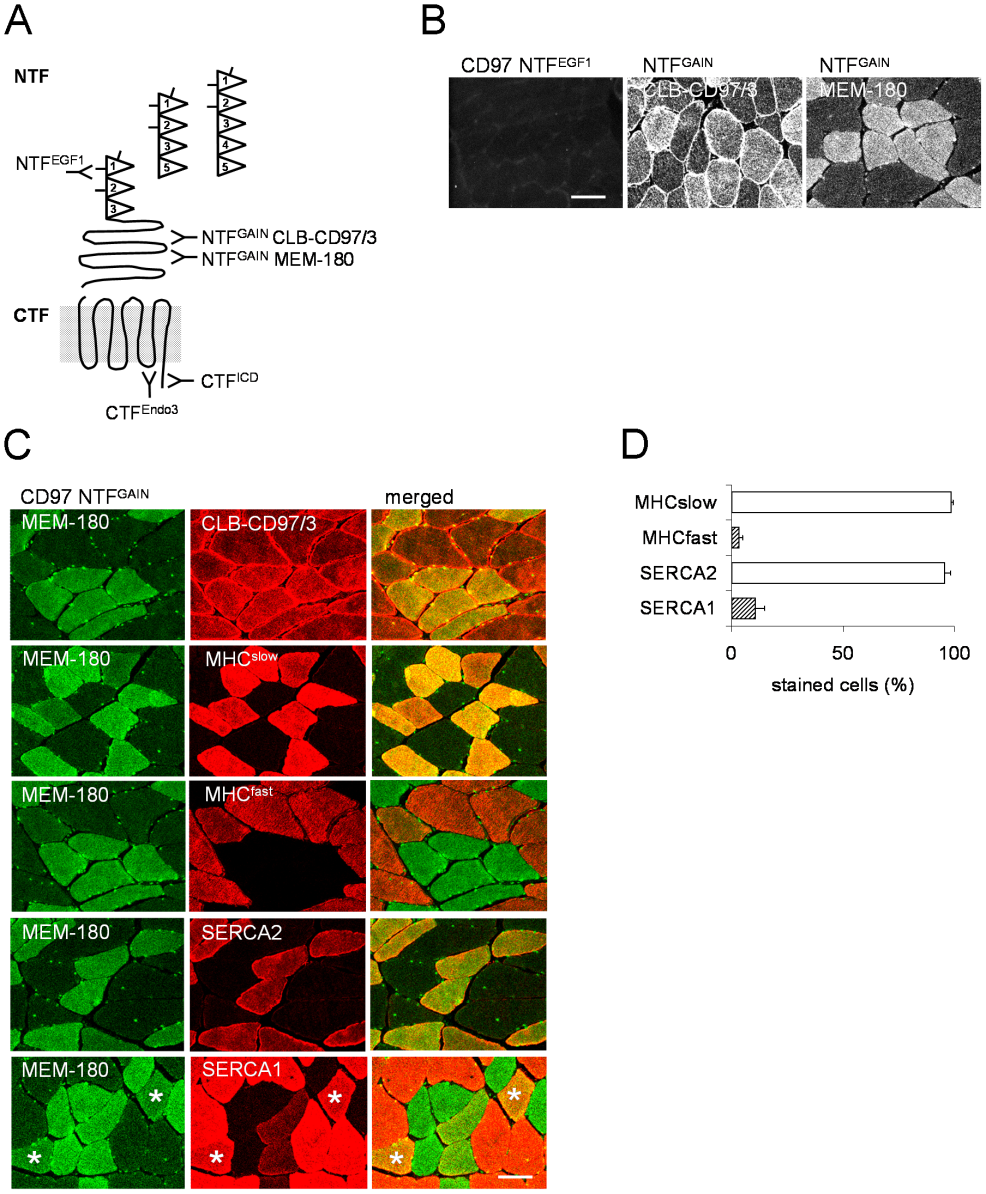
CD97 is expressed in human skeletal muscle. **A** Schematic presentation of the human CD97 isoforms containing three (EGF125), four (EGF1235), or five (EGF1-5) EGF-like domains in the NTF [10]. Indicated are the binding sites of the Abs used in this study. The exact binding site of all NTF^GAIN^ Abs within the large GAIN-domain is unknown. **B** The CD97 NTF^EGF1^ Ab BL-Ac(F2), detecting an N-glycosylation-dependent epitope in the first EGF-like domains, did not bind to normal human skeletal muscle. In contrast, the sarcolemma of all fibers was stained by the NTF^GAIN^ Ab CLB-CD97/3. The NTF^GAIN^ Abs CLB-CD97/3 and MEM-180, binding to different epitopes within the GAIN domain, intensively stained a subpopulation of muscle fibers intracellularly. **C** Double immunofluorescence staining of a representative vastus medialis muscle. The fibers that were stained more strongly by CLB-CD97/3 and MEM-180 were the same. Co-staining with the MEM-180 and MHC- or SERCA-specific Abs indicated that slow-twitch fibers were more strongly CD97-positive. A small subpopulation of SERCA1-positive myofibers also stained more strongly for CD97 (asterisk). **D** Percentage of MEM-180 strongly-positive fibers, set to 100%, co-stained with MHC- and SERCA-specific Abs (n = 170 fibers, mean ± SEM).

The specificity of the Abs used for the detection of human (hCD97) and mouse (mCD97) CD97 is illustrated in figure 1A, in the mouse section, and summarized in table 1. Each CD97 Ab is restricted in its binding to the NTF or CTF, its species specificity, and/or its application. For clarity, the Abs used in this study are indicated in the text together with their binding site.

**Table 1:** CD97 antibodies used in this study.

Binding site	Clone name/Catalog number	Reference/Company	Immunized with/Comments	Specificity	Origin	Application
NTF^EGF1^	BL-Ac(F2)	[5]	Human peripheral blood leukocytes/Epitope N-glycosylation-dependent [19]	human	Mouse, monoclonal	IH, FC
NTF^GAIN^	CLB-CD97/3	[34]	hCD97(EGF125)-transfected cells	human	Mouse, monoclonal	IH, FC
NTF^GAIN^	MEM-180	[33], Immunotools	PHA-activated human peripheral blood lymphocytes	human	mouse, monoclonal	IH
NTF^GAIN^	HPA013707	Sigma	90-aa polypeptide from GAIN domain of hCD97	human	Rabbit, polyclonal	WB
CTF^Endo3^	ab13345	Abcam	Polypeptide from 3^rd^ endoplasmatic loop of hCD97	human	Rabbit, polyclonal	IH
CTF^ICD^	-	[9]	13 most C-terminal aa of mCD97	human, mouse	Rabbit, polyclonal	WB
NTF^EGF1/2^	1B2	[48]	mCD97(1234)-transfected cells	mouse	Armenian hamster, monoclonal	FC
NTF	AF3734	R&D Systems GmbH (AF3734)	aa 24–477 of mCD97(EGF124)	mouse	goat	WB

amino acid (aa), flow cytometry (FC), immunohistology (IH), phytohemagglutinin (PHA), Western blotting (WB).

Abs with other specificities were purchased as follows: myosin heavy chain (fast type, MHC^fast^; slow type, MHC^slow^) from Novocastra Laboratories (Newcastle upon Tyne, UK), desmin (cloneD33) and myogenin (clone F5D) from DakoCytomation (Hamburg, Germany), α-actinin and α-tubulin from Sigma-Aldrich (Taufkirchen, Germany), RyR1 (clone C334), SERCA1 (clone VE121G9), SERCA2 (clone IID8), and DHPR (clone 1A) from Novus Biochemicals (Cambridge, UK), smooth muscle actin (SMA, clone ASM-1) from Progen (Freiburg, Germany), and muscle actin (clone HHF35) from Enzo Diagnostics (Farmingdale, NY, USA). The goat SERCA2 polyclonal Ab was from Santa Cruz Biotechnology (Heidelberg, Germany). The myomesin monoclonal Ab was a kind gift from Dr. R. Schoenauer (Institute of Cell Biology, ETH Hönggerberg, Zürich).

### Murine skeletal muscle morphometry and myofiber typing

For morphometric analysis and fiber typing, murine skeletal muscles were prepared and weighted (n = 10 mice/strain, 10–12 weeks old). In hematoxylin-eosin (HE)-stained cross sections, the muscles were compared for cross-sectional area, number of fibers/area, and cross-sectional area of fibers. Fibers were typed into slow- and fast-twitch according to their MHC isoform either by double immunofluorescence or by immunohistology. Metabolic fiber typing into slow oxidative, fast oxidative glycolytic, and fast glycolytic is based on immunoreactivity of slow and fast MHC monoclonal Abs, and activities of succinate dehydrogenase (SDH), a marker of oxidative activity, and glycerol-3-phosphate-dehydrogenase (GPDH), a marker of glycolytic activity, in serial cross sections of the same fibers as described [24]. To demonstrate activity of myofibrillar adenosine triphosphatase (ATPase) in murine muscles, the method of Punkt et al. [25] was slightly modified by using an acid preincubation solution with a pH 4.45.

### Real-time PCR

Relative gene expression levels were measured using ABsolute qPCR Sybr Green Mix (Abgene, Epsom, UK) on a Rotor Gene 3000 thermal cycler (Qiagen, Hilden, Germany) with the cycle threshold method. Expression of specific genes was normalized to murine RPS29 [26] as endogenous control, using the primers (forward/reverse) 5′-tgaaggcaagatgggtcac-3′/5′-gcacatgttcagcccgtatt-3′. For mCD97, the primer pair 5′-ggtgccctatcaactcaaaatgtg-3′/5′-caggtgtcgtcccagtgttca-3′ was used.

### Laser scanning microscopy

Serial cryostat sections were cut to 6 µm, fixed in 4% paraformaldehyde for 10 min, and incubated with the primary Ab at 4°C overnight. Primary mouse monoclonal Abs were detected with chicken anti-mouse Ab F(ab)_2_ fragment and primary goat or rabbit polyclonal Abs with rabbit anti-goat or goat-anti-rabbit Abs (all Life Technologies, Darmstadt, Germany) for 1 h. The secondary Abs were labeled either with Alexa Fluor-488 or -546. Double labeling was done by combining Abs from mouse, rabbit, goat, and chicken to prevent unspecific binding. Slides were analyzed by laser-scanning microscopy (LSM5 Pascal; Carl Zeiss, Jena, Germany).

### Immunohistochemistry

Serial cryostat sections of rhabomyosarcomas were cut to 6 µm, fixed in ice-cold acetone for 10 min, and incubated with the primary Ab or with an isotype control of irrelevant specificity (mouse IgG1; R&D Systems Wiesbaden-Nordenstadt, Germany) at 4°C overnight. Primary Abs were detected with a biotinylated rabbit anti-mouse-Ig or goat-anti-rabbit-Ig (Vector Laboratories, Burlingame, CA, USA) for 30 min, followed by incubation with a peroxidase-labelled avidin-biotin-complex (Vector) for 1 h. The enzyme was visualized with 3, 3′-diaminobenzidine (DAB; Sigma).

### Flow cytometry

Normal human skeletal muscle cells and Hs729.T cells were stained with the primary CD97 monoclonal Abs for 30 min at 4°C, followed by phycoerythin-labeled goat-anti-mouse polyclonal Ab (Life Technologies) for 20 min at 4°C. For CD97 detection in C2C12 cells, the biotinylated CD97^NTF^ Ab 1B2 was detected by streptavidin-phycoerythin (Life Technologies). Cells were fixed with 1% paraformaldehyde and analyzed by flow cytometry.

### Biochemical characterization of CD97

Tissue protein lysates were prepared in T-PER tissue protein extraction reagent (Thermo Fisher Scientific, Dreieich, Germany). Lysates of 1 × 10^7^ cells were prepared in M-PER mammalian cell protein extraction reagent (Thermo). A total of 2–20 µg protein was separated on a 7.5% SDS polyacrylamide gel and transferred to nitrocellulose, which was probed with the NFT^GAIN^ Ab HPA013707 and a CTF^ICD^ Ab to detect hCD97. For mCD97, the transferred proteins were probed with the NFT^GAIN^ Ab AF3734 and the CTF^ICD^ Ab. IRDye 680-coupled goat-anti-rabbit or donkey-anti-goat secondary Abs were used (LI-COR Biosciences, Bad Homburg, Germany). Blots were analyzed with the Odyssey CLx (LI-COR).

### Preparation of isolated sarcolemmal membrane vesicles

Skeletal muscles of WT mice were rapidly excised and kept in 0.75 M KCl, 5 mM imidazole, pH 7.4 at 4°C. Sarcolemmal membrane vesicles were obtained by centrifugation on a discontinuous Dextran T10 density gradient as described [27].

### Electron microscopy

Rectus femoris muscle subtly dissected from left quadriceps femoris muscle, the lateral part of the triceps brachii muscle, and the diaphragm from 4 WT and 4 CD97Ko mice aged 4 months were carefully prepared. Samples minced into small blocks of about 1 mm^3^ were prefixed in 2.5% glutaraldehyde/0.1 M cacodylate buffer and postfixed in 2% OsO_4_/0.1 M cacodylate buffer. After overnight en bloc stain with saturated uranyl acetate, the specimens were dehydrated and embedded in Durcupan ACM (Sigma). Thin sections were analyzed with a Zeiss EM 900 electron microscope (Oberkochen, Germany). For ultrastructural morphometry, we analyzed 25 electron micrographs at 20,000-fold primary magnification obtained from five different tissue blocks per muscle. For each micrograph, the following structures were sized: diameter of SR and triads, length of sarcomers, and ratio of triads and AJ-junctions per 100 µm^2^ myofibrillar area. Myocyte cytoplasmic volume densities were determined in a blinded fashion using the principles of Weibel [28]. Data were expressed as volume density (volume of subcellular structure [μm^3^] per cytoplasmic volume [μm^3^] of the myofibrils, SR, T-tubules, and sarcoplasm).

### Isolation of intact flexor digitorum brevis (FDB) myofibers

FDB muscles were dissected from 15-16-weeks old WT or CD97Ko male mice and incubated in DMEM with 4.5 g/l glucose and 0.11 g/l sodium pyruvate (Life Technologies). After removing connective tissue and blood vessels, FDB muscles were incubated with 0.2% collagenase (Worthington Biochemical Corporation, Lakewood, NJ, USA) in DMEM/10% fetal calf serum at 37°C for 90 min. Muscle bundles were transferred to DMEM/10% fetal calf serum. Intact single myofibers were isolated from bundles by flipping the tube and trituration with Pasteur pipettes of decreasing diameters and from broken fibers by gravity sedimentation by passing through three medium columns. Myofibers were seeded on 13-mm cover slips coated with 0.57 µg/μl laminin (Sigma) as described [29] and cultured at 37°C for 5 h.

### Intracellular Ca^2+^ measurements

Myofibers were incubated with DMEM containing 2.5 µM Fura-2AM and 0.01% pluronic F-127 (Life Technologies, Darmstadt, Germany) for 45 min in the dark and then washed in DMEM for 20 min. The coverslip was placed in an RC-26G laminar flow imaging chamber (Warner Instruments LLC, Hamden, CT, USA) on the stage of an inverted IX-71 microscope, equipped for fluorescence (Olympus, Hamburg, Germany). The chamber with a 234-μl bath volume was perfused by gravity at a flow of 1 ml/min. One to three fibers with intact sarcolemmal membranes and regular striation patterns were selected per single experiment. All myofibers were loaded with 20 µM N-benzyl-p-toluenesulfonamide (BTS; Calbiochem, Darmstadt, Germany) in DMEM for 15 min to prevent motion artifacts from muscle contraction [30]. To induce Ca^2+^ release, either 30 mM caffeine (Sigma) or 100 mM KCl (Calbiochem) were added. 5 µM ionomycin (Life Technologies) was applied as a positive control at last. Images were recorded every 2 s for 5 min. Fluorescence images at 340 nm and 380 nm excitation were recorded at each time point and 340/380 nm ratio of fluorescence intensity was calculated after correction for background and bleaching using a TILLvisION 4.5 data acquisition software and analysis package (TillPhotonics, Gräfelfing Germany) [31].

### Functional assessment of soleus muscles

Soleus muscle of male WT (n = 8) and CD97ko (n = 8) mice at the age 2 and 4 months were used for functional assessment by determining the force–frequency relationship in an organ bath. The muscles were incubated in an oxygenated (95% O_2,_ 5% CO_2_) physiological buffer containing (in mmol/l) 120.5 NaCl, 4.8 KCl, 1.2 MgSO_4_, 1.2 NaH_2_PO_4_, 20.4 NaHCO_3_, 1.6 CaCl_2_, 10 dextrose, and 1 pyruvate (pH 7.4) at 30°C. The isolated muscle was fixed with a silk suture at the proximal and distal tendons, and mounted in an organ bath for the measurement of muscle contractile function (Aurora Scientific, Aurora, Ontario, Canada) essentially as described [32]. In brief, the resting tension of the soleus muscle was continuously monitored and adjusted, if necessary, to 2.0 *g* throughout the measurement. Under the stretched condition, the muscle was left quiescent for 15 min and was then subjected to a series of three isometric twitches (1 Hz), followed by three tetani (125 Hz) spaced 1 min apart, to elicit consistent contractile responses before any additional experimental procedures were performed. After an additional 5-min quiescent period, the stretched muscle was subjected to a force–frequency protocol at electrical stimulation frequencies of 25, 50, 75, 100, 125, and 150 Hz, each for a duration of 200 ms and spaced 1 min apart. After the force–frequency protocol, a protocol to measure muscle fatigue was added. The regime consisted of a tetanus (100 Hz), which was applied for 15 s. Both maximal and minimal forces were measured, and the decline over time was calculated. Results were adjusted to a muscle cross-sectional area, which was calculated as recently described [32].

### Mouse activity wheels

We investigated wheel-running activity of WT and CD97Ko mice to gain insight into physical effort. 16 mice of each strain (8 female, 8 male) at the age of 8 weeks were housed for 24 h every 14 days in cages containing activity wheels (Intellibio, Nomeny, France). Wheels were connected to a system that used a magnetic sensor to record wheel rotations, which was interfaced with a computer. The mice had free access to the wheel. The following parameters were recorded: speed and acceleration (mean and maximum), duration of running (mean/run and over 24 h), and distance (mean/run and over 24 h).

### Statistics

The following statistical tests were applied: Mann-Whitney test, Student's *t-* and Chi quadrate test. Statistical analyses were performed using SPSS version 20.0 (IBM Deutschland, Ehningen, Germany). Data are provided as mean ± SEM. *P* values less than 5% were considered as significant.

## Results

### Intracellular expression of CD97 in human slow-twitch fibers

A couple of established Abs binding to the NTF [5], [33], [34] were used to localize CD97 in human skeletal muscle (fig. 1A). In cross sections, staining failed with the CD97 NTF^EGF1^ Ab BL-Ac(F2) (fig. 1B), indicating that CD97 is not or only partially N-glycosylated within its EGF-like domains in normal skeletal muscle. The CD97 NTF^GAIN^ Ab CLB-CD97/3, but not MEM-180, stained the peripheral sarcolemma of all myofibers strongly (fig. 1B). Additionally, individual fibers displayed different intracellular staining: a fiber subpopulation expressed CD97 at a moderate level, whereas others showed only a faint or even no intracellular staining (fig. 1B). This difference was most obvious with the NTF^GAIN^ Ab MEM-180.

To examine which fiber type more strongly expresses CD97, skeletal muscles were co-stained for CD97 together with MHC and SERCA. Both NTF^GAIN^ Abs, CLB-CD97/3 and MEM-180, labeled the same fibers (fig. 1C). MHC^slow^-positive fibers were more strongly stained by MEM-180 compared to most MHC^fast^-positive fibers (fig. 1C, D). Only a few MHC^fast^-positive fibers expressed CD97 at a moderate level. Examination of cross sections (n = 50-180 fibers/muscle) in vastus mediales (n = 7), semitendinosus (n = 3), and spine muscles (n = 5) revealed CD97 expression in 3.9±1.9%, 1.9±1.6%, and 4.2±2.0% (mean ± SEM) of the MHC^fast^ -positive cells.

### CD97 co-localizes with SERCA in longitudinal sections

**Figure 2:**
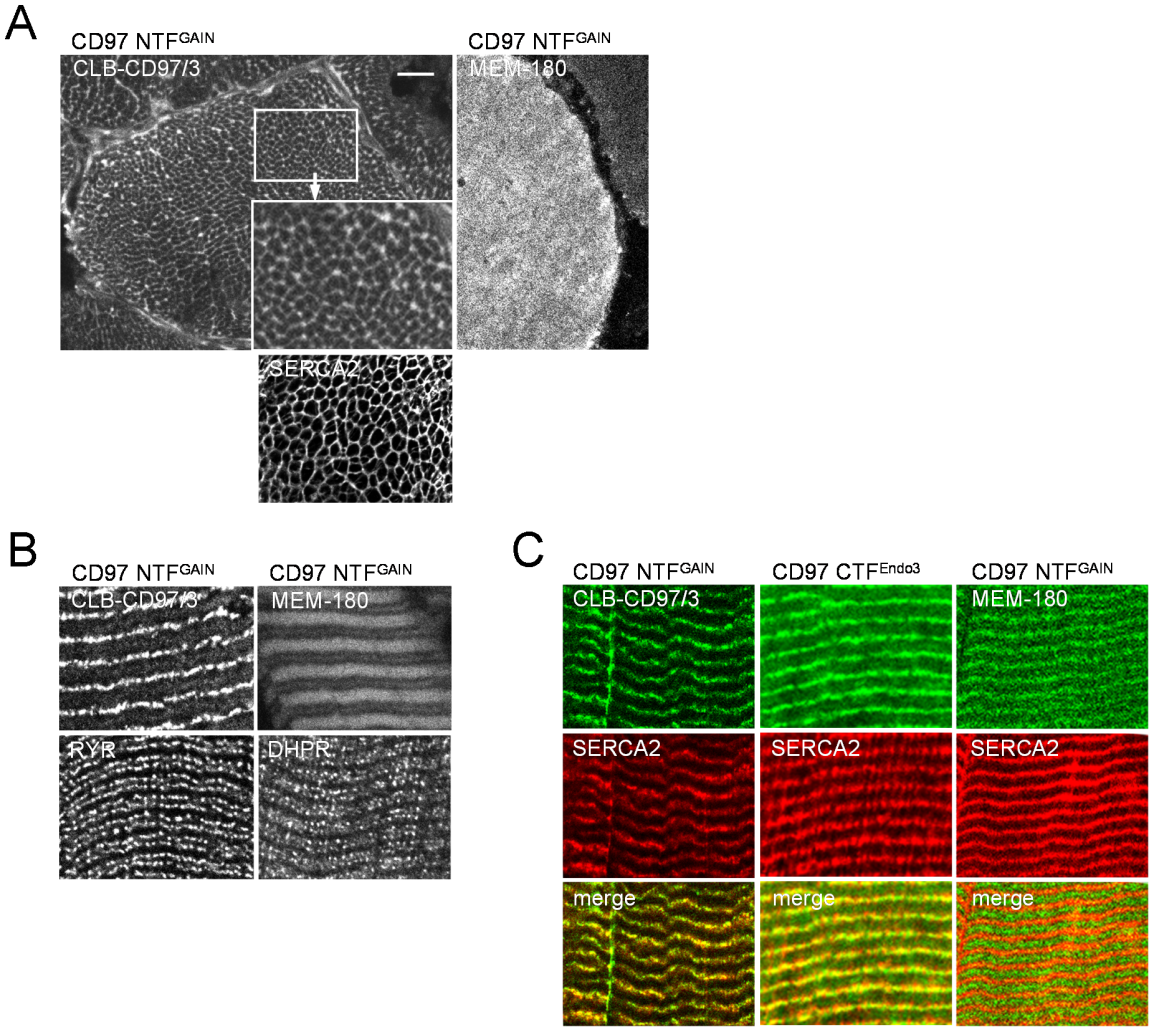
CD97 co-localizes with SERCA. **A** In cross-sections, CD97 NTF^GAIN^ CLB-CD97/3 and SERCA2 Abs showed a honeycomb-like staining pattern, indicating localization of CD97 within the SR or T-tubules (scale bar 10 µm). A blurred, fuzzy staining was seen with the CD97 NTF^GAIN^ Ab MEM-180 inside the fibers. **B** In longitudinal sections, a striated pattern was seen with CLB-CD97/3 and MEM-180, whereas staining for RYR and DHPR yielded two parallel dotted lines. **C** The CD97 Abs CLB-CD97/3 and NTF^Endo3^ ab13345 co-localized with SERCA2 in longitudinal sections, whereas the MEM-180 projected to the M-band.

At higher magnification, we observed with CD97 Abs directed against NTF^GAIN^ (CLB-CD97/3) (fig. 2A) and CTF^Endo3^ (ab13345; data not shown) a polygonal, honeycomb-like staining pattern within the fibers, indicating that the full-length receptor is present inside the cell. The honeycomb-like pattern is typical for proteins with SR or T-tubule localization, such as SERCA, RYR, and DHPR. In contrast, intracellular staining with the NTF^GAIN^ Ab MEM-180 looked blurred and fuzzy (fig. 2A).

To localize CD97 correctly in the SR or T-tubules, longitudinal sections were examined. Staining with the NTF^GAIN^ Ab CLB-CD97/3 revealed a striated pattern consisting of an alternating thick and a very faint, sometimes invisible thin band (fig. 2B). None of the CD97 Abs showed a dotted, close double band typical for staining of the DHPR or the RyR, thus, excluding localization of CD97 in T-tubules or in the terminal cisternae of the SR. The NTF^GAIN^ Ab CLB-CD97/3 and the CTF^Endo3^ Ab co-localized with SERCA2 confirming the feasible location of CD97 in the longitudinal SR (fig. 2C). In contrast, the thick NTF^GAIN^ Ab MEM-180-positive band projected to the M-band. The staining patterns of the different CD97 Abs in cross and longitudinal human skeletal muscle sections are summarized in table 2.

**Table 2:** CD97 staining pattern in normal human skeletal muscle.

Epitope (antibody)		NTF^GAIN^ (CLB-CD97/3)	NTF^GAIN^ (MEM-180)	CTF^Endo3^ (ab13345)
Immunohistology		positive	positive	positive
Cross-sections	honey-comb like staining pattern	x	-	x
	discrimination between fiber types	weak	strong	weak
	sarcolemma	2+	±	±
Longitudinal sections	striated staining pattern	x	x	x
	bands	alternately one thick and one ultrathin band	alternately one thick and one thin band	alternately one thick and one ultrathin band
	projection to	thick band: Z-line	thick band: M-line	thick band: Z-line
Thick band: colocalization with	RyR	-	-	-
	DHPR	-	-	-
	SERCA	x	-	x
	myomesin	-	x	-

The CD97 NTF^EGF1^ Ab BL-Ac(F2) stained malignant but not normal skeletal muscle and thus is not provided here.

### Rhabdomyosarcomas strongly express CD97

**Figure 3:**
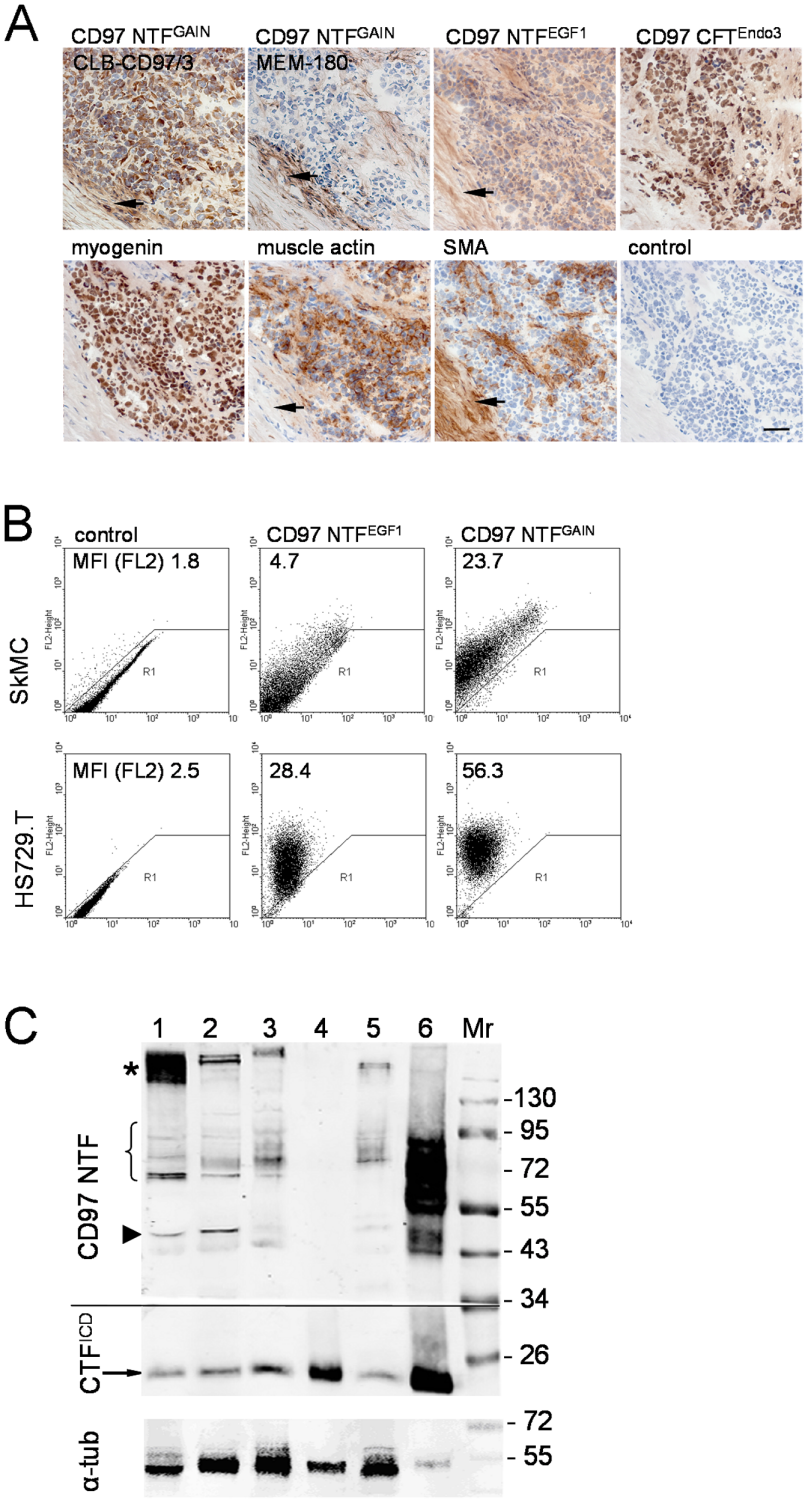
Rhabomyosarcoma strongly express CD97. **A** Immunostaining of a rhabomyosarcoma (patient no. 1, table 3; alveolar subtype). The tumor is positive for myogenin and muscle actin (HHF35) but negative for smooth muscle actin (SMA), typical for most rhabdomyosarcomas. Tumor cells were strongly stained by the NTF^GAIN^ CLB-CD97/3 and NTF^Endo3^ ab13345 Abs. The NTF^EGF1^ Ab BL-Ac(F2) showed weak staining, indicating partial N-glycosylation of the CD97 EGF-like domains. The NTF^GAIN^ Ab MEM-180 did not stain tumor cells. The tumor infiltrated a smooth muscle which is SMA-positive (arrow) and expresses slightly CD97. Scale bar 50 µm. **B** In flow cytometry, normal human skeletal muscle cells (SkMC) and Hs729.T cells were CLB-CD97/3-positive, whereas the NTF^EGF1^ Ab BL-Ac(F2) only stained the tumor cells more strongly, indicating the presence of N-glycosylated CD97. **C** Western blot analysis using the CD97 NTF^GAIN^ Ab HPA013707 and a CFT^ICD^ Ab. After blotting, the membrane was cut horizontally at a height of 34 kDa. The upper part of the blot was incubated with the CD97 NTF^GAIN^ Ab and the lower part with the CFT^ICD^ Ab. 1: rhabdomyosarcoma, 2: cultured normal human skeletal muscle cells (SkMC), 3: HS729T cells, 4: duodenum of Tg(CD97-villin) mice [16], 5: human peripheral blood lymphoytes, 6: human HT1080 cells overexpressing hCD97(EGF125) [8]. Mr, molecular weight marker. The band of about 50 kDa (arrow head) probably represents the naked protein core of the NTF; it is not present in HS729T and peripheral blood lymphocytes. The bands of 70-98 kDa may represent different hCD97 isoforms or differently glycosylated forms. The rhabdomyosarcoma showed an additional high-molecular weight band (asterisk). With the CTF^ICD^ Ab, a 25-kDa band representing the CTF was detected in all lysates (arrow) including the murine tissue because this Ab detects human as well as murine CD97. Incubating the entire blot with the CTF^ICD^ Ab showed the additional high-molecular weight band in the rhabdomyosarcoma which indicates an uncleaved CD97 form in this tumor (data not shown). In lanes 1-3 and 5, 10 µg, in lane 4, 5 µg, and in lane 6, 2.5 µg protein were separated. Loading was controlled with an α-tubulin Ab, shown in the lower panel.

Rhabdomyosarcomas, the malign tumors of skeletal muscle, can be classified as alveolar or non-alveolar, including embryonal and pleomorphic variants. All examined rhabdomyosarcomas including two pleomorphic cases, which were negative for myogenin, were strongly stained by CD97 Abs against NTF^GAIN^ (CLB-CD97/3) and CTF^Endo3^ (table 3; fig. 3A). This indicates the presence of full-length CD97 in malignant skeletal muscle tumors independent of the subtype. In one patient (no. 4), the recurrent tumor showed stronger CD97 expression compared to the primary one. Interestingly, in the embryonal subtype, only half of the tumor cells expressed myogenin, whereas all were positive for the CLB-CD97/3. In contrast to normal muscle, many of the rhabdomyosarcomas showed staining with the NTF^EGF1^ Ab BL-Ac(F2) (table 3; fig. 3A) suggesting increased N-glycosylation of CD97 within its EGF-like domains. Both pleomorphic tumors expressed this epitope at high level. Surprisingly, all tumors failed to show staining with the NTF^GAIN^ Ab MEM-180, i.e., this epitope was lost during tumorigenesis.

**Table 3:** Expression of CD97, muscle actin, and myogenin in rhabdomyosarcomas of six patients.

No	Sex	Age	Grading	TNM	Stage	Subtype	CD97 epitope (antibody)^1^				Muscle actin	Myogenin
							NTF^EGF1^ (BL-Ac(F2))	NTF^GAIN^ (CLB-CD97/3)	NTF^GAIN^ (MEM-180)	CTF^Endo3^ (ab13345)		
1	m	16	3	pT1bNxMx	II	alveolar	±	4+	0	4+	4+	4+
2	m	72	3	pT2aNxMx	IIB	pleomorphic	2+	4+	1+	4+	0	0
3	m	16	3	pT1aNxMx	IIA	alveolar	±	2+	0	3+	nd	2-3+
4	m	17	3	pT1aNxMX	IIA	alveolar	0	3+	0	3+	2+	2-3+
		18	3	relapse		alveolar	±	4+	0	4+	4+	4+
5	f	2	3	pT2bNxMx	III	embryonal	1+	3+	0	50% 0; 50% 4+	few cells (5%) 4+	50% 0; 50% 4+
6	m	45	3	pT2bNxMx	III	pleomorphic	4+	2+	0	3+	2+	0

Staining intensity of tumor cells was judged as follows: 0: no staining; 1+: weak staining, 2+: moderate staining, 3+: strong staining; and 4+ very strong staining. Percentages indicate that only a fraction of tumor cells stained positive.

1 CD97 was stained with Abs directed against different epitopes. Tumor, node, metastasis (TNM), not determined (nd).

Rhabdomyosarcoma Hs729.T cells were positive for CD97 Abs specific for NTF^EGF1^ (BL-Ac(F2)) and NTF^GAIN^ (CLB-CD97/3) in flow cytometry (fig. 3B). Thus, in these tumor cells most CD97 is N-glycosylated, which was confirmed by Western blot analysis (fig. 3C). Cultured normal human skeletal muscle cells were more strongly positive for the NTF^GAIN^ Ab CLB-CD97/3 compared to the NTF^EGF1^ Ab BL-Ac(F2). These cells probably express more non-N-glycosylated CD97. Indeed, the NTF^GAIN^ Ab HPA013707 detected a 50-kDa band, presumably representing the unglycosylated CD97 core, and weaker bands around 70-95 kDa indicating only partial N-glycosylation of CD97. In a slightly NTF^EGF1^ (BL-Ac(F2))-positive rhabdomyosarcoma (patient no. 1, table 3), unglycosylated and N-glycosylated CD97 bands could be detected in parallel by Western blotting. Human peripheral blood lymphocytes, used as a positive control, expressed N-glycosylated CD97 only.

### CD97 is present in murine skeletal muscles

**Figure 4:**
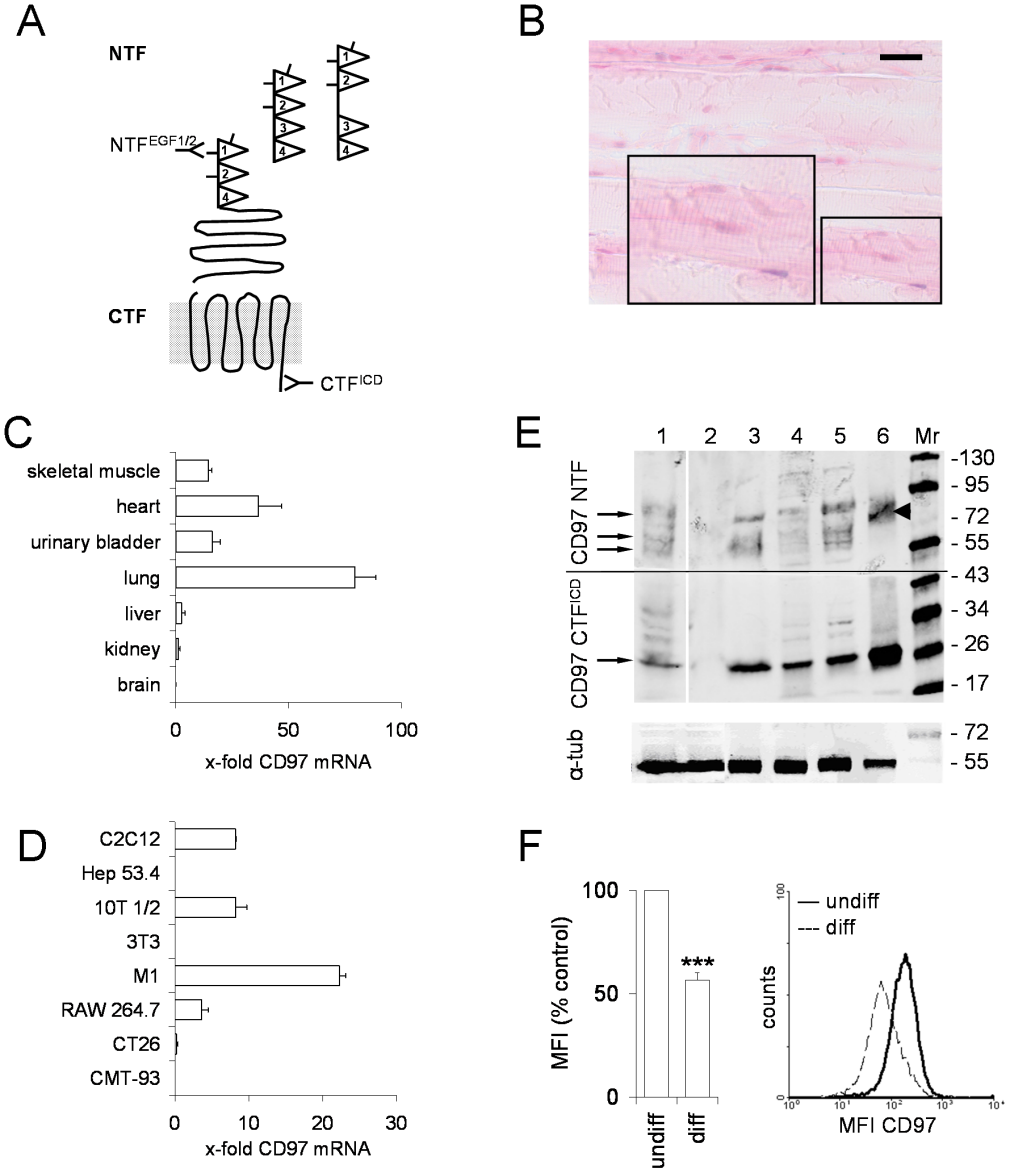
CD97 is present in murine skeletal muscle. **A** Schematic presentation of the murine CD97 isoforms containing three (EGF124), four (EGF1234), or four EGF-like domains plus 45 additional amino acids (EGF12×34) [47]. Indicated are the binding sites of the Abs used in this study. The CD97 NTF Ab AF3734 is not shown because its exact binding site within the NTF is unknown. **B** Nuclear β-galactosidase staining (blue) with fast red counterstaining in the soleus muscle of a CD97-lacZ knock-in mouse; scale bar 50 µm. **C, D** CD97 mRNA levels of murine tissues (C) and cell lines (D) were quantified by RT-PCR. RSP29 normalized log_2_ x-fold mRNA levels compared to brain and CMT-93 cells, which were set to one, are given (n = 3, mean ± SEM). **E** The upper part of the Western blot was incubated with the CD97 NTF Ab AF3734, whereas the lower part was incubated with a CD97 CFT^ICD^ Ab. 1: WT mouse, soleus muscle, 2: CD97Ko mouse, soleus muscle, 3: WT mouse, enriched sarcolemma, 4: C2C12 myocytes cultured in 10% FCS, 5: C2C12 myoblasts cultured in 2% horse serum, 6: positive control duodenum Tg(villin-CD97) mouse overexpressing CD97(EGF1234) in intestinal epithelial cells [16]. In lanes 1–2, 20 µg, in lanes 3–5, 10 µg, and in lane 6, 5 µg protein were separated; Mr: molecular weight standard. Loading was controlled with an α-tubulin Ab, shown in the lower panel. Using the CFT^ICD^ Ab, a 26-kDa band representing the CD97 CTF was present in all samples except the CD97Ko mouse (lane 2). The CD97 NTF AF3734 Ab detected the NTF of the CD97(EGF1234) isoform expressed in the Tg(villin-CD97) mouse (arrow head). Additional NTF bands were present in the WT soleus muscle, the enriched sarcolemma, and the C2C12 lysates, perhaps representing the various CD97 isoforms and/or not N-glycosylated protein of these isoforms (arrows). **F** C2C12 myoblasts (undiff) showed higher levels of cell-surface CD97 compared to differentiated (diff) C2C12 myocytes in flow cytometry (mean fluorescence intensity, MFI; n = 5, mean ± SEM; ***p< 0.001, Mann-Whitney test). The right panel shows a representative flow cytometry plot.

The specificity of the Abs used for the detection of mCD97 is illustrated in figure 4A. Skeletal muscle of CD97-lacZ knock-in mice were analyzed for the expression of β-galactosidase, because detection of mCD97 failed with the only Ab that works in immunohistology [9]. Nuclei of skeletal fibers were slightly positive for β-galactosidase (fig. 4B). Quantification of mCD97 mRNA in skeletal muscle yielded levels comparable to those of smooth muscle-containing tissues such as the urinary bladder (fig. 4C). Western blot analysis revealed the presence of various CD97 isoforms in murine skeletal muscle at low level. The soleus muscle, consisting of 30–40% of slow-twitch fibers [35], showed more CD97 expression compared to the tibialis anterior muscle, mainly consisting of fast-twitch fibers. Enrichment of muscular sarcolemma confirmed the presence of CD97 at the surface of murine skeletal muscle.

Among several murine cell lines, undifferentiated C2C12 myoblasts showed intermediate levels of CD97 mRNA (fig. 4D). The result was confirmed in flow cytometry and Western blot analysis at the protein level (fig. 4E, F). Differentiation of C2C12 myoblasts into myocytes forming contractile myotubes decreased CD97 surface expression by about 50% (fig. 4F).

### CD97-deficient myofibers show a dilated SR

**Figure 5:**
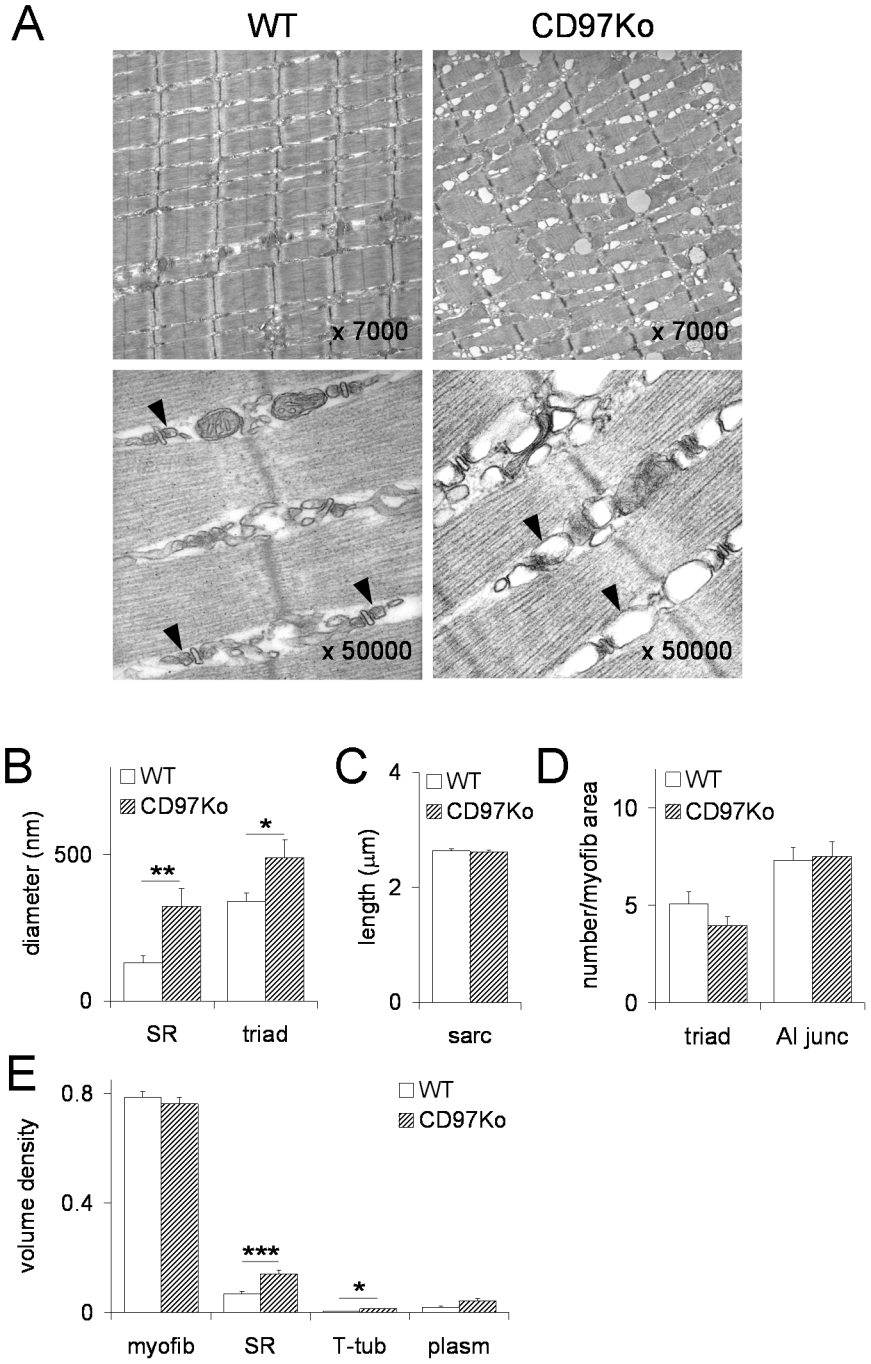
The SR in skeletal muscles of CD97Ko mice is morphologically abnormal. **A** Representative electron micrographs of longitudinal ultrathin sections of WT and CD97Ko mice. Note the altered morphology of the SR which was frequently swollen (arrow heads), whereas myofibrils and mitochondria retained normal structures in CD97Ko mice. **B-E** Quantitative measurements of several morphometric parameters based on electron micrographs of the quadriceps muscle (n = 4 mice/strain, 25 micrographs/animal, mean ± SEM; *p<0.05, **p<0.01, *** p<0.001, t-test). D shows the number of AI-junctions and triads/100 µm^2^ area occupied by myofibrils.

Next, skeletal muscles of CD97Ko mice were examined for ultrastructural changes. Myofibrils and mitochondria retained normal structures. Abnormalities were seen in the SR of CD97-deficient muscles with all fibers being affected. The longitudinal SR regions were partially vacuolated, the terminal SR regions were frequently swollen, and the orientation of the SR networks was irregular (fig. 5A). The observed differences were quantified by morphometric analysis of micrographs, shown here exemplarily for the quadriceps muscle. The diameter of the SR was increased in all CD97-deficient muscles (fig. 5B). The effect was most obvious in the quadriceps (2.48-fold increase in CD97Ko compared to WT) as compared to the triceps (1.67-fold) and diaphragm (1.87-fold). Only in the quadriceps, the diameter of cross-sectioned T-tubules was larger in CD97Ko compared to WT mice. The increased diameter of the SR and T-tubules in this muscle resulted in an increased diameter of the triads (fig. 5B). The length of sarcomers as well as the number of AI-junctions and triads/100 µm^2^ area occupied by myofibrils was comparable between both mice (fig. 5C, D). Volume densities also reflect the observed changes: volume density of the SR was higher in all mutant muscles whereas volume density of myofibrils and sarcoplasm was comparable (fig. 5E). We concluded that the SR of CD97-deficient muscles was dilated without other obvious ultrastructural changes.

**Figure 6:**
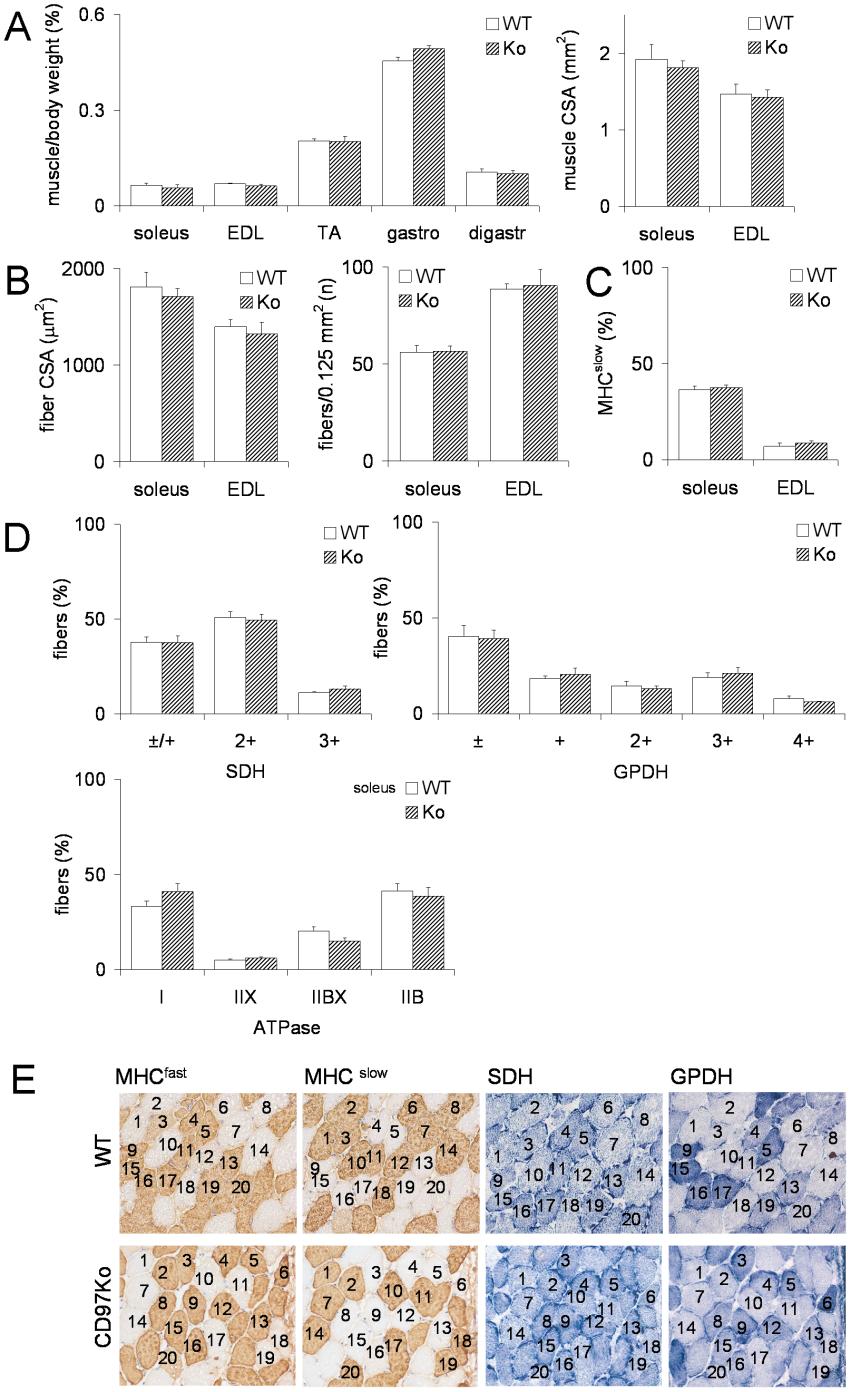
Macroscopic and microscopic features of adult CD97Ko and WT skeletal muscles are comparable. **A-E** 10 mice per strain were analyzed; data are mean ± SEM (t-test). **A** Percentage of muscle to body weight and cross-sectional area (CSA) of several skeletal muscles: extensor digitorum longus (EDL), tibialis anterior (TA), gastrocnemius (gastro), digastricus (digastr). **B** Cross-sectional area (CSA) of single fibers (n = 30/mouse) and number of fibers/area (n = 2 areas/muscle) of soleus and EDL muscles. **C** Percentage of MHC^slow^-positive fibers within the EDL and soleus muscles stained by immunohistology. **D** Histochemistry for succinate dehydrogenase (SDH), glycerol-3-phosphate-dehydrogenase (GPDH), and ATPase (pH 4.5) in soleus muscles. Staining intensities were quantified according to visible subtypes. **E** Physiologic and metabolic profile of murine soleus muscles of a WT and CD97Ko mouse; 20 fibers were identified and typed in each section. WT mouse (fibers 1-3, 6-8, 10, 14, and 18 are MHC^slow^-positive, all fibers have the metabolic phenotype slow-oxidative; fibers 4, 5, 9, 12, 13, 15–17, 19, and 20 are MHC^fast^-positive, metabolic phenotype fast-oxidative glycolytic II; fiber 11 is MHC^fast/slow^-double-positive, metabolic phenotype slow-oxidative); CD97 Ko mouse (fibers 7, 10, 11, 14, and 17–20 are MHC^slow^-positive, metabolic phenotype slow-oxidative; fibers 1, 3–6, 8, 9, 12, 13, 15, and 16 are MHC^fast^-positive, metabolic phenotype fast-oxidative glycolytic II; fiber 2 is MHC^fast/slow^-double-positive, metabolic phenotype slow-oxidative).

Next, we clarified whether CD97-deficient muscles show further morphometric and metabolic abnormalities. We found no differences in i) the percentage of muscle relative to body weight and the muscle cross-sectional area (fig. 6A), ii) the single fiber cross-sectional area and the number of fibers per defined area, i.e., the fiber density (fig. 6B), iii) the distribution and percentage of MHC^slow^- (fig. 6C) and MHC^fast^-positive fibers (not shown), and iv) the distribution and percentage of metabolic fiber types demonstrated by histochemistry for SDH, GPDH, and ATPase (fig. 6D). Representative results of the soleus muscle are shown in figure 6E. For extensor digitorum longus (EDL) muscles, the results of CD97-deficient and WT muscles were also comparable (not shown).

### CD97 does not affect skeletal muscle function in mice

**Figure 7:**
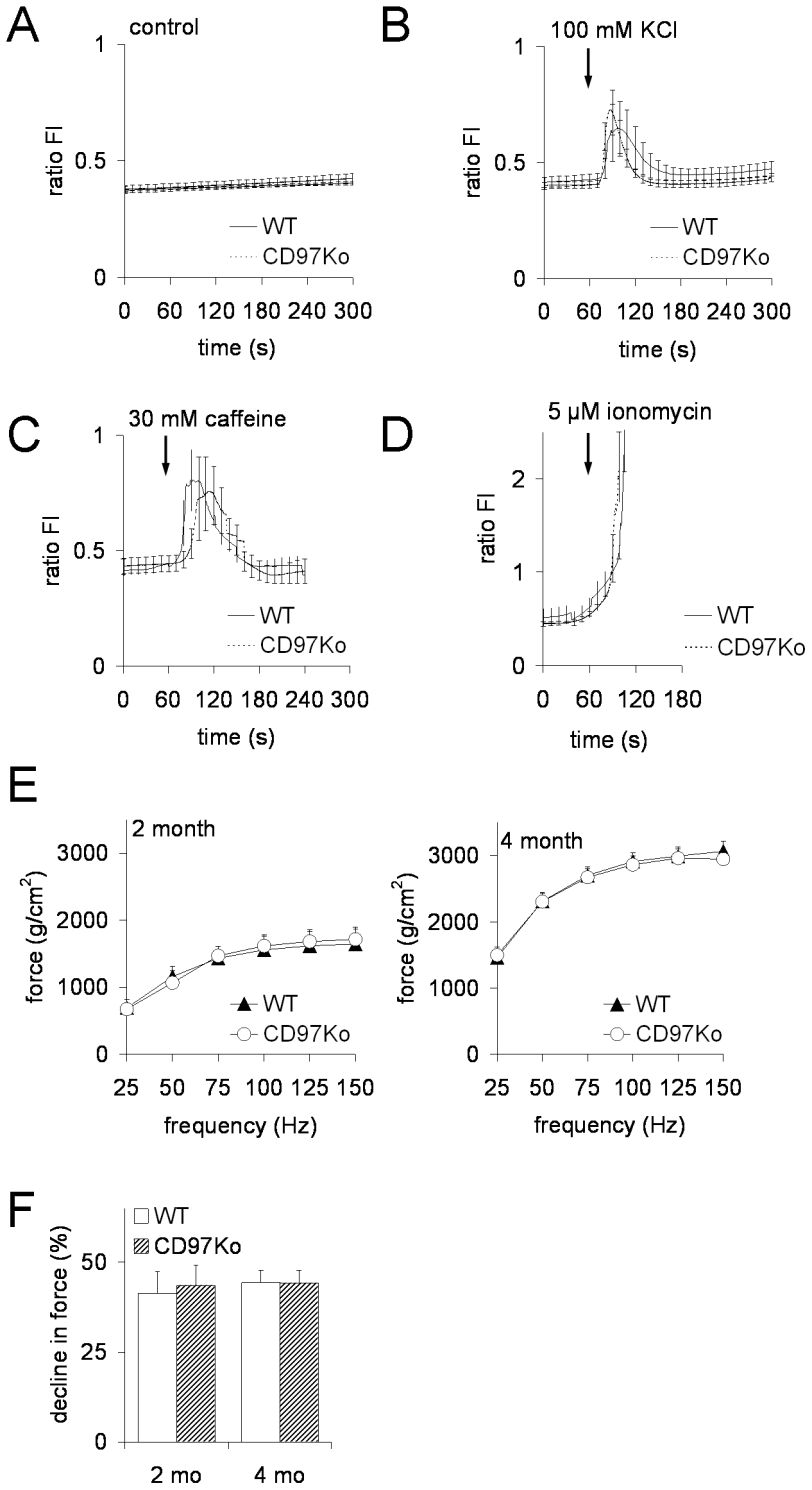
Calcium release from myofibers and muscle force generation and fatigability are normal in CD97Ko mice. **A–D** Intracellular Ca^2+^ release from the SR into cytoplasm of single muscle flexor digitorum brevis (FDB) myofibers. Mean traces of the analyzed FDB fibers from adult WT and CD97Ko represent [Ca^2+^]i responses measured by Fura-2AM video microscopy. Variations in [Ca^2+^]i over time are represented by the ratio fluorescence intensities (FI) at 340 and 380 nm excitation wavelengths after dynamic background subtraction. Fibers were exposed to calcium release activators after 60 s. From 120 s till 300 s, medium without a substance was applied again. 5 mice per strain and from each mouse two to three fibers were analyzed (mean ± SEM, Mann-Whitney test). **A** Basal 340/380 fluorescence intensities (FI) ratios were unchanged in both WT and CD97ko fibers. **B, C** 100 mM KCl (B) and 30 mM caffeine (C) increased [Ca^2+^]i comparably in WT and CD97Ko fibers. **D** Application of 5 µM ionomycin as a positive control induced a fast total store [Ca^2+^]i release from the SR. **E, F** Functional analysis of skeletal muscles of CD97Ko and WT mice at the age of 2 and 4 months. Force-frequency relationship (E) and muscle fatigability, determined as percentage of decline in force over 15 s (F), were measured in soleus muscles (n = 8 mice/strain; mean ± SEM, t-test).

Finally, we examined whether the disturbed SR ultrastructure in CD97-deficient myofibers affects skeletal muscle function. Firstly, we measured the capacity to release intracellular calcium from the SR after application of high potassium and caffeine to induce depolarization in Fura-2-loaded single, intact FDB fibers of WT and CD97Ko mice (Fig. 7A–D). We found no differences in the ratio of basal fluorescence intensities (FI) (Fig. 7A) and in the response to 100 mM KCl (Fig. 7B), 30 mM caffeine (Fig. 7C), or 5 µM ionomycin (Fig. 7D).

Secondly, we assessed force generation and fatigability of the soleus muscle of WT and CD97Ko animals (Fig. 7E, F). As shown in figure 7E, maximal force generation at stimulation frequencies higher than 25 Hz was comparable in 2- and 4-months old mice of both strains. CD97 had no impact on fatigability during a short-term challenge protocol in WT and CD97Ko mice (Fig. 7F).

**Figure 8:**
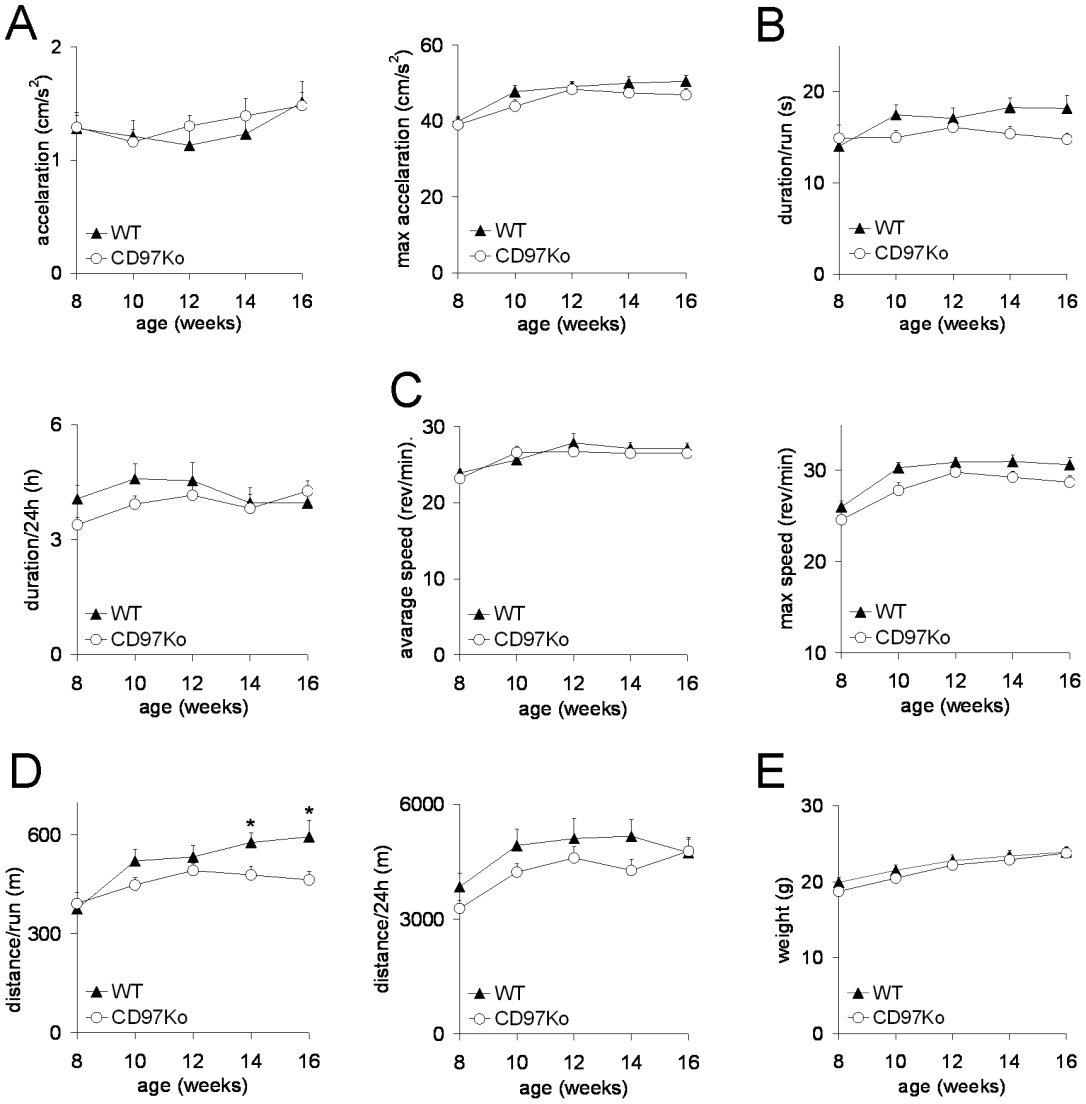
Unchanged wheel-running activity of CD97Ko mice. Measures of wheel-running activity of CD97Ko and WT mice (n = 16 mice/strain; mean ± SEM; t-test, *p<0.05). Running for 24 h was repeated every 2 weeks. **A–E** Average and maximum acceleration (A), duration/run and over 24 h (B), average and maximum speed (C), and distance/run or over 24 h (D) were measured. Body weight was monitored (E).

Thirdly, we compared the wheel-running capacity between CD97Ko and WT mice. For nearly all parameters, especially for maximum running speed, duration per run, and overall running time per 24 h, a slight but not significant decrease in CD97Ko compared to WT mice was observed (fig. 8A–D). The covered distance per run was higher in WT mice at the age of 14 and 16 weeks. Body weight was not affected in parallel over time (Fig. 8E).

## Discussion

We report here that normal human and murine skeletal muscles express the aGPCR CD97. CD97 is present in all myofibers at the peripheral sarcolemma as shown by immunostaining of human tissue sections and by cell surface labeling of human skeletal muscle cells and murine C2C12 cells in flow cytometry. Moreover, in pure sarcolemmal preparations of murine skeletal muscle, the CD97 NTF and CTF were detected by Western blot analysis. In addition to this sarcolemmal expression, CD97 is localized intracellularly. In human skeletal muscle, CD97 is present primarily in slow-twitch myofibers, which enable continuous, extended muscle contractions and fatigue less than fast-twitch fibers. The highly similar staining pattern for both CD97 fragments and SERCA, in cross and longitudinal muscle sections indicates the presence of CD97 in the SR.

Ultrastructural analysis of *CD97*-deficient muscle supports CD97 localization at the SR, as this muscle revealed a dilated and irregular SR structure in the absence of a functional *CD97* gene. Initially, intracellular location of full-length CD97, which is a 7TM receptor with a highly hydrophobic CTF, seems counterintuitive. Probably, CD97 locates not inside the SR, but may be integrated into its membrane. This is supported by our previous findings that CD97 was also detected inside disseminated tumor cells at the invasion front of colorectal carcinomas [8], indicating that the intracellular location of CD97 described here is not restricted to skeletal muscle cells. We earlier obtained evidence that during epithelial–mesenchymal transition, CD97 is translocated from lateral adherens junctions of normal intestinal epithelial cells into the cytoplasm in malignant cells [16].

The dilated and irregular SR could lead to functional defects in skeletal muscles of mice lacking CD97. To test this, WT and CD97-deficient soleus muscles, 30–40% of them having slow-twitch characteristics, were isolated, and maximal force generation at stimulation frequencies higher than 25 Hz and fatigability during a short-term challenge protocol were assessed and found to be comparable. These data were confirmed by intracellular Ca^2+^-release experiments. High potassium, caffeine, and ionomycin were applied to single WT and CD97-deficient FDB myofibers, but no difference was seen. Perhaps the FDB muscle, mainly consisting of fast-twitch fibers, was not optimal for Ca^2+^-release experiments. However, the extraordinary long soleus myofibers [35] are not suitable for Ca^2+^-release experiments. Consistent with the other functional data, we observed that the wheel-running activity of CD97Ko mice was comparable to that of WT mice. Forced-running experiments with mice were not scheduled because the lung epithelium is strongly CD97-positive [9]. Thus, forced-running activity may be influenced additionally by the CD97-deficiency in the lung.

Despite clear ultrastructural changes in CD97-deficient muscles, functional readouts yielded no obvious differences in skeletal muscle function. This finding is similar to other models, where the loss of SR-resident proteins, thought to have major roles in Ca^2+^ handling, produces ultrastructural defects without causing major functional impairment. For example, SERCA2 plays a significant role in muscle contraction by pumping Ca^2+^ from the cytosol into the SR, allowing for muscle relaxation and refilling of the SR with reasonable Ca^2+^ [22]. However, mice with a targeted disruption of *Serca2* in skeletal muscle are viable, and basic muscular (ultra)structure was intact with only a reduction in the relative longitudinal SR volume [36]. And although muscle relaxation was slowed, maximal force was maintained. Furthermore, skeletal muscles lacking sarcalumenin, one of the luminal Ca^2+^-binding proteins in the longitudinal SR [22], showed an irregular SR. Nevertheless, *Srl1*-deficient mice are normal in growth, health, and reproduction [37], and their skeletal muscles retained normal mechanisms of excitation–contraction and bear only partially impaired signaling after contraction [37]. Thus, loss of proteins located in the SR can result in marked ultrastructural changes which are compensated functionally.

Our data indicate that CD97 contributes to the structure of the SR, although the molecular function of CD97 remains unclear. In two hypothesis-free microarray studies, CD97 was found to be regulated in skeletal muscle. First, in differentiated C2C12 myotubes, high K^+^-induced depolarization over 5 min caused *CD97* mRNA downregulation after 24 h, indicating CD97 involvement in the adaptive response after skeletal muscle stimulation [38]. Second, *CD97* mRNA was upregulated in skeletal muscles of women with the metabolic syndrome and correlated with insulin resistance [39].

There are indications that CD97 is related to skeletal muscle differentiation. First, CD97 is reduced at the surface of C2C12 cells, when switched from growth to differentiation medium, which stimulates the transition of myoblasts to myocytes. Indeed, the general function of aGPCRs in skeletal muscle may relate to differentiation. Very recently, two class members, BAI1 and GPR56, were shown to be involved in myogenesis. BAI1 prompted the fusion of mononucleated C2C12 myoblasts to multinucleated skeletal myofibers via binding to phosphatidylserin [40]. GPR56 was upregulated during early differentiation of C2C12 cells, and myoblasts lacking GPR56 showed decreased fusion *in vitro* [41]. However, as mice lacking CD97, GPR56-deficient mice had no overt skeletal muscle phenotype [42]. The second indication for an association of CD97 with skeletal muscle differentiation comes from the rhabdomyosarcomas studied here. In contrast to normal skeletal muscle, all tumors were strongly positive for the NTF^GAIN^ Ab CLB-CD97/3, i.e., during dedifferentiation, CD97 expression increased.

Interestingly, CD97 was present in all subtypes of rhabdomyosarcomas and thus seems to be closely associated with this malignant tumor type, perhaps even more than muscle-specific actin and desmin [43], [44]. However, as a biomarker, CD97 lacks specificity, because other neoplasms including leiomyosarcomas also express CD97 [21]. Tumorigenesis of skeletal muscle was accompanied in part by an increase of N-glycosylated CD97, as suggested by staining with the CD97 NTF^EGF1^ Ab BL-Ac(F2) and by Western blotting. This finding closely matches our previous observation in malignant smooth muscle [19]. As in rhabomyosarcomas, staining of CD97, now mostly N-glycosylated, with BL-Ac(F2) increased in leiomyosarcomas.

Overall, the expression of CD97 in normal and malignant human skeletal muscle is complex. Although both CLB-CD97/3 and MEM-180 bind within the 285-amino acid GAIN domain that is located between the 7TM region and the EGF-like domains, the partially different staining pattern indicates that they detect different epitopes with restricted (intra)cellular localization. In cross-sections both Abs stained slow-twitch fibers more strongly compared to most fast-twitch fibers, yet MEM-180 stained the sarcolemma only slightly, did not provide the typical honeycomb-like pattern in cross-sections, and projected not to the SERCA band in longitudinal sections, which means that this epitope is present inside myofibers, but not in the SR. The blurry staining pattern of MEM-180 in cross-sections rather indicates that this epitope is probably located at or between the myofibrils, which are enveloped by the SR/T-tubule system. Thus, the CLB-CD97/3 and MEM-180 epitopes are located at different places within the same myofiber and are not present at the same CD97 molecule.

CD97 exists in various isoforms [10], different glycosylation states [19], membrane-bound as well as soluble forms [45], and it is located at the cell-surface but also intracellularly [8]. Moreover, Hsiao et al. demonstrated the presence of partially uncleaved CD97 in overexpressing cells *in vitro* [46]. We obtained indications that also *in vivo* uncleaved CD97 is present: Western blotting of a rhabdomyosaroma yielded an additional high molecular weight band using the NTF^GAIN^ HPA013707 and the CTF^ICD^ Abs (data not shown). The complexity of the various CD97 forms is probably the reason for the staining patterns found with different, well-established monoclonal Abs in skeletal muscle. Whether these different staining and thus location pattern of the NTF^GAIN^ Abs CLB-CD97/3 and MEM-180 are related to any of the existing CD97 forms could not be clarified in the present study because suitable Abs are missing.

The difference between both epitopes was most obvious in rhabdomyosarcomas: All tumor cells were strongly NTF^GAIN^ CLB-CD97/3-positive but MEM-180-negative. The disappearance of the MEM-180 epitope parallels our own results on the presence of CD97 in smooth muscle [21]. In leiomyosarcomas, we observed that some of these malignant tumors completely lost the epitope detected by the CLB-CD97/3 Ab although normal smooth muscle cells express this epitope [21]. In contrast, in rhabdomyosarcomas selectively the epitope recognized by MEM-180 is missing.

In summary, CD97 is located at the peripheral sarcolemma and in the SR of human skeletal muscle. Absence of its expression in mice causes a dilated SR without leading to an obvious impairment in skeletal muscle function.
